# Differential vulnerability of hippocampal CA3-CA1 synapses to Aβ

**DOI:** 10.1186/s40478-022-01350-7

**Published:** 2022-04-04

**Authors:** Olivia A. Shipton, Clara S. Tang, Ole Paulsen, Mariana Vargas-Caballero

**Affiliations:** 1 Department of Physiology, Development and Neuroscience, University of Cambridge, Cambridge, CB2 3EG UK; 2 Department of Physiology, Anatomy and Genetics, OXION Initiative, University of Oxford, Oxford, OX1 3PT UK; 3 School of Biological Sciences, Faculty of Environmental and Life Sciences, University of Southampton, Southampton, SO17 1BJ UK

**Keywords:** Alzheimer’s disease, Synapse, Tau, Asymmetry, Hippocampus, Optogenetics

## Abstract

Amyloid-beta (Aβ) and tau protein are both involved in the pathogenesis of Alzheimer’s disease. Aβ produces synaptic deficits in wild-type mice that are not seen in *Mapt*^−/−^ mice, suggesting that tau protein is required for these effects of Aβ. However, whether some synapses are more selectively affected and what factors may determine synaptic vulnerability to Aβ are poorly understood. Here we first observed that burst timing-dependent long-term potentiation (b-LTP) in hippocampal CA3-CA1 synapses, which requires GluN2B subunit-containing NMDA receptors (NMDARs), was inhibited by human Aβ_1–42_ (hAβ) in wild-type (WT) mice, but not in tau-knockout (*Mapt*^*−/−*^*)* mice. We then tested whether NMDAR currents were affected by hAβ; we found that hAβ reduced the postsynaptic NMDAR current in WT mice but not in *Mapt*^*−/−*^ mice, while the NMDAR current was reduced to a similar extent by the GluN2B-selective NMDAR antagonist Ro 25–6981. To further investigate a possible difference in GluN2B-containing NMDARs in *Mapt*^*−/−*^ mice, we used optogenetics to compare NMDAR/AMPAR ratio of EPSCs in CA1 synapses with input from left vs right CA3. It was previously reported in WT mice that hippocampal synapses in CA1 that receive input from the left CA3 display a higher NMDAR charge transfer and a higher Ro-sensitivity than synapses in CA1 that receive input from the right CA3. Here we observed the same pattern in *Mapt*^*−/−*^ mice, thus differential NMDAR subunit expression does not explain the difference in hAβ effect on LTP. Finally, we asked whether synapses with left vs right CA3 input are differentially affected by hAβ in WT mice. We found that NMDAR current in synapses with input from the left CA3 were reduced while synapses with input from the right CA3 were unaffected by acute hAβ exposure. These results suggest that hippocampal CA3-CA1 synapses with presynaptic axon originating in the left CA3 are selectively vulnerable to Aβ and that a genetic knock out of tau protein protects them from Aβ synaptotoxicity.

## Introduction

Alzheimer’s disease (AD) is characterized by an accumulation of oligomeric amyloid beta (Aβ) and misfolded and mislocalized microtubule-associated protein tau (MAPT; tau protein). These pathological features are thought to trigger synaptic failure, followed by progressive synaptic and neuronal loss [1]. The hippocampus is one of the first brain regions affected in AD [2], and the number of synapses is already halved in the hippocampal CA1 region in patients with mild AD [3]. This early loss of synapses suggests that synaptic dysfunction is an important contributor to cognitive impairment in AD patients. Understanding the initial pathological changes at the synapse will be helpful in developing therapeutic strategies to prevent neural circuit dysfunction.

Acute or chronic exposure to Aβ causes a deficit in long-term potentiation (LTP) at CA3-CA1 hippocampal synapses in rodents [4–6]. This impairment is an early functional indicator of failing synapses [7] and also provides a useful model in which to study changes that could impair cognitive function since LTP is thought to support long-term memory [8]. Indeed, a reduction in LTP magnitude correlates with cognitive impairments in transgenic animal disease models, which exhibit LTP deficits when memory impairments are already detectable [6, 9].

Whilst changes in synaptic strength are likely important for cognitive function, synapses do not all show the same capacity for such plasticity. For example, clear differences exist in the mouse hippocampus where selective recruitment of the left or right CA3 input to the CA1 using optogenetics revealed that the left CA3 input to CA1 synapses shows burst timing-dependent LTP (b-LTP), whilst the right CA3 input to CA1 synapses does not, irrespective of ipsilateral vs contralateral location of postsynaptic CA1 response recordings [10]. This striking dissociation in LTP magnitude also extends to LTP induced by high frequency stimulation, with CA1 synapses receiving left CA3 input potentiating more than those receiving right CA3 input [11].

The left–right asymmetry in hippocampal LTP is explained by differences in postsynaptic spines on CA1 neurons. The majority of spines on CA1 neurons receiving input from the left CA3 are morphologically ‘thin’ and rich in GluN2B subunit-containing NMDA receptors (NMDARs) [10, 12, 13]. PSD area size and spine head volume correlate with the presynaptic origin of CA3 fibres (left or right hippocampus) but not with their ipsilateral or contralateral origin [13]. Thin spines have a higher turnover rate but can be strengthened and stabilized by the addition of AMPA receptors (AMPARs) and enlarge following an LTP protocol [14]. Furthermore, single spine imaging has shown that the GluN2B subunit-selective NMDAR antagonist Ro 25–6981 reduces glutamate uncaging-evoked EPSCs and Ca^2+^ transients only in small spines [15]. In contrast, the less plastic projection from the right CA3 tends to synapse with larger mushroom-shaped postsynaptic CA1 spines with a lower density of GluN2B subunit-containing NMDARs and a higher density of AMPARs [10, 12, 13]. Mushroom spines have been considered more mature spines, since they can be stable for months [16–18] and show no permanent morphological changes following an LTP protocol [14].

The different molecular, morphological and plastic properties of these two main types of spines have led to the proposal that they make different contributions to cognitive function, with thin spines being responsible for the acquisition of new information whilst large spines represent permanent memory traces that are resistant to disruption [19, 20]. The hemispheric asymmetry in spine populations correlates with long-term memory performance; optogenetic inhibition of the left CA3, which is the source of the more plastic inputs to CA1 in both left and right hippocampus, impairs long-term memory- whilst silencing the right CA3 does not [10, 11]. Consequently, maintaining a functional population of thin spines during adulthood might be vital to continually acquire new information, and pathological changes to, or loss of, such spines might therefore cause cognitive deficits.

To understand more about the processes leading to synaptic failure in AD, we assessed whether acute application of Aβ differentially affects these two synapse populations with different plastic properties. We used wild-type (WT) and *Mapt*^*−/−*^ mice, which have comparable basal synaptic properties and CA3-CA1 tetanus-induced NMDAR-dependent LTP [21]. We found that NMDAR-mediated currents were reduced by Aβ only in CA3-CA1 synapses with input from the left CA3 in WT mice and not in CA3-CA1 synapses in *Mapt*^*−/−*^ mice, despite an asymmetric distribution of GluN2B subunit-containing NMDARs also in these tau-knockout mice. If synapses with distinct morphological and molecular characteristics are differentially vulnerable during disease progression, this may reveal novel cognitive impairment mechanisms and additional therapeutic targets in AD.

## Results

### Aβ inhibits burst timing-dependent LTP in wild-type mice and lack of tau protein prevents this effect

**Fig. 1:**
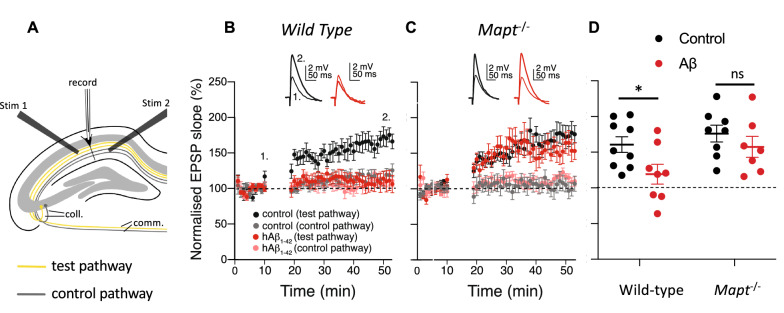
Human Aβ_1-42_ blocks b-LTP in wild-type, but not *Mapt*^*−/−*^ mice. **a** EPSPs were evoked with two electrical stimulation electrodes (stim 1 and stim 2). Each electrode activated an independent pathway (test or control pathway, respectively) of hippocampal Schaffer collateral (coll.) and commissural (comm.) projections to CA1 pyramidal neurons in WT mice (**b**) and *Mapt*^*−/−*^ mice (**c**). After a 10-min baseline, the burst timing-dependent plasticity protocol was applied to one of these pathways (test pathway; black and red symbols) while the other pathway served as control (control pathway; gray and pink symbols). Both test and control pathways were monitored for a further 35 min. Recordings were made in aCSF following incubation for 1–3 h in 220 nM hAβ_1-42_ (hAβ_1-42_; red and pink) or vehicle control (control; black and gray). Representative EPSP traces are from the test pathway in hAβ_1-42_ (red) and control aCSF (black) at the indicated time points (1. Baseline, prior to the induction protocol, 2. At the end of the recording). **d** The mean of the normalized EPSP slope during the last five minutes of recording (30–35 min post-pairing; mean for control pathway not shown) was used as the outcome measure. Following two-way ANOVA (see text), significance was tested with post-hoc corrected Student’s *t*-tests. Error bars are SEM. *P < 0.05

Burst timing-dependent LTP (b-LTP) [22] is induced in the adult rodent hippocampus by pairing a presynaptic spike with a postsynaptic current injection that elicits a burst of action potentials within a precise time window [23]; b-LTP is completely blocked by GluN2B subunit-selective NMDAR antagonists and is expressed solely at CA3-CA1 synapses with input from the left CA3 [10]. We first wanted to test whether this form of LTP is sensitive to Aβ in WT mice. We made whole-cell current-clamp recordings from CA1 pyramidal cells in mouse hippocampal slices. Using extracellular stimulating electrodes to elicit excitatory postsynaptic potentials (EPSPs) alternately in two independent pathways, we applied a burst pairing protocol to one (test) pathway whilst monitoring specificity and stability of recording in the other (control) pathway (Fig. 1a). These experiments were performed with the experimenter blind to the treatment of the slices with either oligomeric human Aβ_1–42_ (hAβ) or vehicle control. We compared the magnitude of b-LTP following incubation with 220 nM hAβ and under control conditions in both WT and *Mapt*^*−/−*^ mice in interleaved experiments and compared the effect of genotype and hAβ exposure on b-LTP magnitude (two-way ANOVA: genotype: F_(1, 28)_ = 4.45, P = 0.044, hAβ exposure: F_(1, 28)_ = 5.56, p = 0.026; Fig. 1b–d). Whereas hAβ elicited the predicted deficit in b-LTP in WT mice (P = 0.024; Fig. 1b, d), there was no significant difference between hAβ and control conditions in *Mapt*^*−/−*^ mice (P = 0.27; Fig. 1c, d).

We considered whether there was a dissociation between genotypes in the effect of hAβ on presynaptic function. To this end, we measured excitatory postsynaptic currents (EPSCs) at − 70 mV in voltage clamp and compared the paired pulse ratio (PPR) between WT and *Mapt*^*−/−*^ mice in control conditions and following Aβ incubation. We did not observe significant differences in PPR between genotypes and conditions (WT control: 2.01 ± 0.23, *n* = 10; *Mapt*^*−/−*^ control: 1.81 ± 0.15, *n* = 8; WT hAβ: 1.97 ± 0.16, *n* = 10; *Mapt*^*−/−*^ hAβ: 2.00 ± 0.18, *n* = 8; two-way ANOVA: no effect of genotype F_1,32_ = 0.19, P = 0.67; no effect of treatment F_1,32_ = 0.13, P = 0.72), meaning there was no evidence for a change in presynaptic properties between WT and *Mapt*^*−/−*^ mice in the presence of hAβ that might contribute to the b-LTP dissociation we observed.

### Aβ reduces GluN2B subunit-containing NMDAR current in WT but not tau-knockout mice

**Fig. 2:**
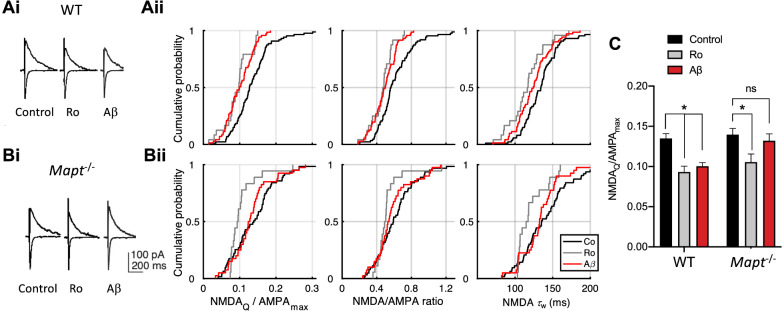
Human Aβ_1-42_ changes NMDAR contribution in wild-type but not *Mapt*^*−/−*^ mice. NMDAR/AMPAR contribution to the EPSC at CA3-CA1 synapses following electrical stimulation under control conditions (black) and following incubation with Ro 25–6981 (gray) or Aβ (red). Representative traces for WT (Ai) and *Mapt*^*−/−*^ (Bi) mice and cumulative distribution plots from wild-type (Aii) and *Mapt*^*−/−*^ mice (Bii). Each point in the cumulative distribution plot shows average value per cell for: charge transfer (NMDA_Q_/AMPA_max_), N/A ratio, and weighted NMDAR current decay time constant (τ_w_). **c** Summary graph (NMDA_Q_/AMPA_max_) to facilitate comparison between WT and *Mapt*^*−/−*^ mice. Following two-way ANOVA (see text), significance was tested with post-hoc corrected Student’s *t*-tests to explore main effects, see also Table 1

Our observations of b-LTP impairment by Aβ together with the absence of presynaptic dysfunction suggest that Aβ may impair postsynaptic function. Since the number and composition of postsynaptic glutamate receptors is important for both effective synaptic transmission and the induction and expression of plasticity, we first investigated the contribution that NMDA and AMPA receptors make to the EPSC. Since Aβ inhibited b-LTP in WT but not *Mapt*^−/−^ mice and this form of plasticity requires GluN2B subunit-containing NMDAR function [10], we measured NMDAR and AMPAR-mediated currents in WT and *Mapt*^*−/−*^ mice using a GluN2B subunit-selective NMDAR antagonist (Ro 25–6981, 0.5 μM) to quantify their postsynaptic contribution in each genotype with and without Aβ (Fig. 2).

To this end, we made whole-cell voltage-clamp recordings from CA1 pyramidal neurons and evoked EPSCs by electrical stimulation, alternating between negative and positive holding potentials to measure the contribution of AMPAR and NMDAR-mediated currents to the evoked EPSC. To estimate the contribution of NMDAR subtypes to these synaptic currents we used three inter-related measures: the NMDAR charge transfer, NMDAR/AMPAR current ratio (N/A ratio) and decay kinetics of the NMDAR current (Fig. 2). Since acute incubation with hAβ does not affect basal CA3-CA1 transmission mediated by AMPARs in WT or *Mapt*^*−/−*^ mice under the same experimental conditions [21], we used the peak AMPAR-mediated current to normalize the NMDAR contribution to the EPSC. Firstly, we measured the normalized NMDAR charge transfer (NMDA_Q/_AMPA_peak_). Secondly, we obtained the NMDAR/AMPAR_peak_ ratio (N/A ratio, average NMDAR current measured 55–57 ms after stimulation, at a time when the fast AMPAR-mediated component of the current had decayed to less than 5% of its peak value) [24, 25]. Thirdly, we measured the weighted decay time constant of the NMDAR current (τ_w_). Together, these measures and the use of Ro 25–6981 allowed us to estimate the NMDAR/AMPAR contribution and assess the GluN2B-mediated component.

Slices were incubated with the GluN2B subunit-selective NMDAR antagonist Ro 25–6981 (0.5 μM) or oligomeric hAβ (220 nM) [26]. As expected, the NMDA_Q_/AMPA_max_, N/A ratio, and τ_w_ were all reduced following treatment with Ro 25–6981 in WT mice (Fig. 2a, Table 1). We observed the largest effect size with the charge transfer measure (NMDA_Q_/AMPA_max_), which was reduced by 32% ± 7.3%. Aβ caused a similar reduction in charge transfer in WT hippocampal synapses (26% ± 5.8%). We then analyzed the effect of Ro 25–6981 on synaptic currents in *Mapt*^*−/−*^ mice. We observed a significant reduction of NMDA_Q_/AMPA_max_ (24% ± 9.6% reduction) and τ_w_ (from 135 ± 2.7 ms to 116 ± 4.9 ms) after Ro 25–6981 incubation, however, there was no significant effect of Aβ on any of the outcome measures in *Mapt*^*−/−*^ mice (Fig. 2b, Table 1). Figure 2c shows a side-by-side comparison of NMDA_Q_/AMPA_max_ in WT and *Mapt *^*−/−*^ mice [two-way ANOVA: main effect of genotype (F_2,297_ = 5.51, P = 0.019), and main effect of treatment (F_2,297_ = 10.80, P < 0.001)] showing the effects of Ro 25–6981 in both genotypes but effect of Aβ only in WT mice. The striking lack of significant effect of Aβ in *Mapt*^*−/−*^ mice suggests a possible mechanistic explanation for why LTP is not vulnerable to the effects of Aβ in these knockout mice. GluN2B subunit-containing NMDARs support a slower NMDAR current than GluN2A subunit-containing NMDARs [21] which results in enhanced charge transfer (i.e. larger NMDA_Q_,AMPA_peak_), a larger N/A ratio and a slower weighted decay time constant (τ_w_). The reduced NMDAR current we observed in WT but not in *Mapt*^*−/−*^ mice after exposure to Aβ suggests a specific targeting of GluN2B subunit-containing NMDARs. Since these properties were not affected by Aβ in *Mapt*^*−/−*^ mice, it raises the possibility that Aβ might impair the synaptic localization of GluN2B subunit-containing NMDARs specifically through a tau-dependent mechanism. This hypothesis is consistent with our findings above (Fig. 1) that following Aβ exposure, a GluN2B subunit-dependent form of plasticity is affected in WT but not in *Mapt*^*−/−*^ mice.

**Table 1:** Summary of charge transfer (NMDA_Q_/AMPA_max_), N/A ratio, and NMDAR weighted decay time constant (τ_w_)

Condition	N	NMDA_Q_/AMPA_max_	± SEM	P value	N/A ratio	± SEM	P value	τ (w)	± SEM	P value
Electrical stimulation of Schaffer collateral/commissural projections										
WT Con	17	0.135	0.006		0.62	0.02		134.7	2.75	
WT Ro	18	0.093	0.007	**0.003**	0.46	0.03	**0.003**	115.8	4.87	**0.003**
WT Aβ	46	0.100	0.005	**0.003**	0.49	0.02	** < 0.001**	124.4	2.72	**0.013**
*Mapt*^*−/−*^ Con	33	0.140	0.008		0.62	0.03		142.5	5.22	
*Mapt*^*−/−*^ Ro	48	0.106	0.010	**0.032**	0.53	0.04	0.108	121.0	4.51	**0.037**
*Mapt*^*−/−*^ Aβ	40	0.132	0.008	0.368	0.58	0.03	0.292	135.5	5.53	0.292
Optical stimulation of left (L) or right (R) hippocampal CA3 projections										
*Mapt*^*−/−*^ L Con	8	0.183	0.027		0.74	0.08		146.1	16.56	
*Mapt*^*−/−*^ L Ro	9	0.066	0.014	**0.003**	0.42	0.03	**0.003**	92.8	7.02	**0.011**
*Mapt*^*−/−*^ R Con	11	0.116	0.016		0.62	0.05		116.1	8.84	
*Mapt*^*−/−*^ R Ro	11	0.120	0.017	0.490	0.62	0.09	0.490	114.4	10.17	0.490
WT L Con	29	0.147	0.010		0.68	0.04		125.4	3.76	
WT L Aβ	31	0.116	0.010	**0.032**	0.54	0.04	**0.018**	123.8	5.75	0.490
WT R Con	31	0.169	0.012		0.77	0.06		134.5	3.64	
WT R Aβ	36	0.162	0.008	0.380	0.73	0.03	0.380	132.4	3.94	0.490

P-values show Student’s *t*-test comparing control condition and drug condition following Benjamini-Hochberg correction. Significant P values (< 0.05) are indicated in bold

### Left–right synaptic asymmetry in tau-knockout mice

To investigate whether synapses are differentially vulnerable to the Aβ-induced deficit in LTP, we utilized the synaptic population targeted by the left CA3 input vs the right CA3 input in the mouse hippocampus as introduced above. Specifically, in WT mice, CA3-CA1 synapses receiving input from the left CA3 are richer in GluN2B subunit-containing NMDARs and are more plastic, whereas synapses targeted by the right CA3 have a lower density of GluN2B subunit-containing NMDARs and are less plastic [10–13].

From our findings above, synaptic NMDAR charge transfer, N/A ratio and weighted time constant are reduced after Aβ exposure in WT mice. These changes in postsynaptic glutamate receptors would be expected to have an effect on LTP, and thus are likely candidates for the initial Aβ-induced synaptic changes that impair LTP (Fig. 1). We hypothesized that GluN2B-rich synapses targeted by the left CA3 are specifically affected by Aβ thus causing the reduction in synaptic NMDA receptor contribution we observed in WT mice. However, before testing this hypothesis we first needed to test whether *Mapt*^*−/−*^ mice have a similar left–right difference in their synaptic populations as that seen in WT mice, characterized by increased sensitivity to Ro 25–6981 (0.5 μM) in CA3-CA1 synapses targeted by the left CA3 compared to the right CA3 [10].

**Fig. 3:**
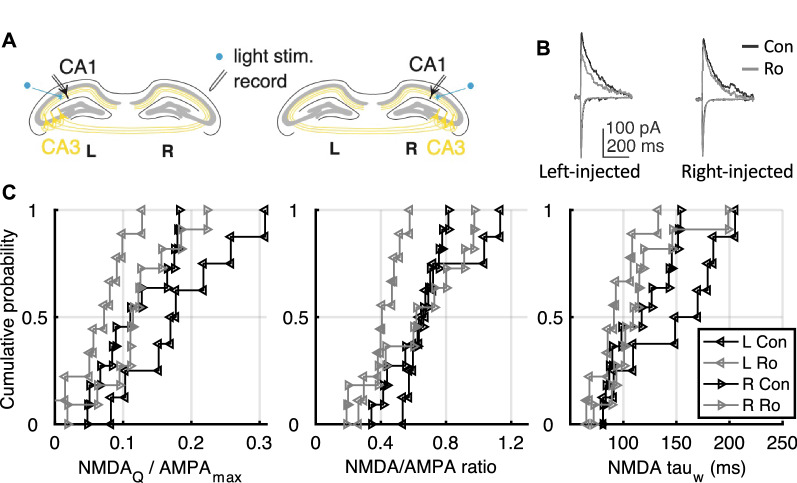
Left/right difference of synaptic NMDARs in *Mapt*^*−/−*^ recordings. **a** Diagram showing selective optical activation of ChR2-expressing hippocampal Schaffer collateral/commissural projections to CA1 pyramidal neurons originating in either the left or right CA3. **b** Representative traces for AMPAR/NMDAR-mediated currents (control condition: black, after GluN2B inhibitor Ro 25–6981: gray) in *Mapt*^*−/−*^ mice expressing ChR2 in left or right CA3. **c** Cumulative distribution plots for charge transfer (NMDA_Q_/AMPA_max_), N/A ratio, and weighted NMDAR current decay time constant (τ_w_) in left or right injected *Mapt*^*−/−*^ mice with and without Ro 25–6981. Statistical comparisons are presented in Table 1

To target the different populations of excitatory CA3-CA1 synapses, we injected an adeno-associated viral vector containing a channelrhodopsin-2 construct (AAV-ChR2) under the control of a CaMKIIα promoter into either the left or right CA3 of adult *Mapt*^*−/−*^ mice (Fig. 3a; for details, see “Methods”). Six weeks after unilateral injection of the construct we used optogenetic stimulation to selectively recruit CA3-CA1 synapses originating in either the left or right CA3, recorded in either the left or right hippocampus. We obtained measures of NMDAR charge transfer, N/A ratio and τ_w_ as described above. We then statistically tested the NMDA_Q_/AMPA_max_ results for effects of hemisphere-injection, hemisphere-recording (ipsilateral/contralateral) and Ro 25–6981 treatment using three-way ANOVA. We did not observe an effect of hemisphere injection on any of the three measures (hemisphere injected F_1,32_ = 0.33, P = 0.57) but we did observe an effect of Ro 25–6981 and also an interaction between the hemisphere injected and Ro 25–6981 exposure (Ro 25–6981 exposure: F_1,32_ = 7.37, P = 0.01; interaction between hemisphere injected and Aβ exposure: F_1,32_ = 7.82, P = 0.008). We did not observe ipsilateral/contralateral effects or interaction between ipsilateral/contralateral hemisphere and drug (hemisphere recorded: F_1,32_ = 0.93 P = 0.34; interaction between hemisphere recorded and Ro 25–6981 exposure: F_1,32_ = 0.82 P = 0.37). We proceeded to test whether Ro 25–6981 reduced the outcome measures (NMDA_Q_/AMPA_max,_ N/A ratio or τ_w_) within each hemisphere (Table 1).

Following treatment with Ro 25–6981, charge transfer was reduced by 64% ± 15.5% in left-injected *Mapt*^*−/−*^ mice but no significant reduction was observed in right-injected *Mapt*^*−/−*^ mice (Fig. 3b, c, Table 1). Likewise, both N/A ratio and τ_w_ were specifically affected in left-injected mice (N/A ratio reduced by 43% ± 9.7% and τ_w_ reduced by 36.5% ± 10.9%, Fig. 3b-c, Table 1). In contrast, we did not see significant differences in any of these measures in right-injected mice. The similar left–right difference in sensitivity to GluN2B antagonist with WT mice [10] indicates that tau-knockout mice have a similar hemispheric dissociation of the two synaptic populations as that seen in WT mice and thus the protective effect in *Mapt*^*−/−*^ mice cannot be explained by an overt difference in the left/right organization of CA3 inputs.

### Left–right asymmetry in synaptic vulnerability to Aβ in WT mice

Finally, we tested the hypothesis that CA1 synapses targeted by the left CA3 are specifically affected by Aβ thus causing the reduction in overall synaptic NMDAR contribution we observed in WT mice. We injected AAV-ChR2 under the control of a CaMKIIα promoter into either the left or right CA3 of adult WT mice, and six weeks after injection we used optogenetic stimulation as above to separate the contribution of left and right CA3 presynaptic inputs to CA1 recorded in either the left or hippocampus (Table 2). We then statistically tested the NMDAR_Q_/AMPA_max_ values for effects of hemisphere injection, hemisphere recording (ipsilateral/contralateral) and Aβ treatment with a three-way ANOVA. We observed an effect of hemisphere injection and an effect of Aβ (hemisphere injected: F_1,120_ = 11.24, P = 0.001; Aβ: F_1,120_ = 4.5, P = 0.03); we did not observe an effect of hemisphere recording (hemisphere recorded: F_1,120_ = 0.67 P = 0.41). We proceeded to test whether Aβ reduced the outcome measures (NMDA_Q_/AMPA_max,_ N/A ratio or τ_w_) within each hemisphere (Table 1).

**Table 2:** Number of cells recorded for each condition. Indicating hemisphere-injected with AAV-CHR2 → hemisphere recorded

*Mapt*-/-	Injected → recorded	N	WT	Injected → recorded	N
Control	L → L	3	Control	L → L	17
	L → R	5		L → R	12
	R → R	2		R → R	26
	R → L	9		R → L	5
Ro 25–6981	L → L	6	Aβ	L → L	23
	L → R	3		L → R	8
	R → R	5		R → R	17
	R → L	6		R → L	19

In mice in which we recruited EPSCs at CA3-CA1 synapses with afferents originating in the left hemisphere (left CA3-injected mice) the charge transfer decreased by 21.1% ± 9.8% following incubation in Aβ, and the N/A ratio decreased by 20.6% ± 8.4% (Fig. 3, Table 1). However, we did not observe a significant change in τ_w_, which could suggest that the number of receptor-channels was reduced by Aβ without a major change in receptor composition.

Remarkably, synapses that originated in the right CA3 (right CA3-injected mice), showed no significant change in any of the measures following hAβ application. This dissociation in the effect of hAβ on postsynaptic NMDARs indicates that these two different synapse populations are differentially vulnerable to Aβ.

## Discussion

We found a differential vulnerability of WT synapses to Aβ, with hippocampal CA3-CA1 synapses with input from the left CA3 showing a reduction in NMDAR current, but no change in those receiving right CA3 input. Our data suggest that GluN2B-containing NMDARs in CA3-CA1 synapses with CA3 axons originating in the left hippocampus are susceptible to Aβ in a tau-dependent manner. The reduction in NMDAR contribution we observed in WT mice was not present in mice that lack tau protein (*Mapt*^*−/−*^ mice), although they do have functional GluN2B subunit-containing NMDARs that show left–right asymmetry. Given that Aβ impairs b-LTP in WT but not in *Mapt*^*−/−*^ mice, a specific tau-dependent reduction in GluN2B subunit-containing NMDARs appears to account for the Aβ-induced impairment of b-LTP.

The hemispheric asymmetry in Aβ-induced pathophysiological changes at the synapse is consistent with the theoretical proposal of ‘molecular nexopathies’ [27], wherein certain neural pathways are particularly vulnerable to the effects of protein abnormalities and this accounts for the unique progression of distinct neurodegenerative diseases. Given that different types of synapse have been proposed to perform distinct functions in learning and memory [28, 29], this may have implications for cognitive function. Specifically, the increase in size following an LTP protocol is transient in large spines while it is sustained in small spines [14], and large spines can be stable for months in the adult [16, 17], leading to the suggestion that thin spines could be particularly important for learning, whilst mature spines could represent a more permanent memory trace [18]. Indeed, in the adult mouse hippocampus, the average spine head volume of CA1 spines receiving input from the right CA3 is 70% larger than those receiving left CA3 input [13], and the left CA3 input potentiates more than the right CA3 input [10, 11]. Furthermore, this synaptic left–right asymmetry may have implications for learning, since optogenetic silencing of the left CA3 or axons from left CA3 pyramidal cells in CA1 during acquisition of a spatial associative long-term memory task impaired performance, but right CA3 silencing had no effect [11, 30]. Patients with mild to moderate AD exhibit a loss of synapses, but the remaining synapses are enlarged so that the total synaptic contact area per unit volume is retained [31], including in the CA1 [3]. If small spines are particularly important for learning, this may help explain why the increased size of remaining spines cannot compensate and certain cognitive functions still become impaired. Although it is not yet known whether humans have an equivalent synaptic asymmetry to that in mice, interestingly, the abnormalities in tissue volume and microstructure that predict the progression from mild cognitive impairment to Alzheimer’s disease first appear in the left hippocampus [32].

To investigate why synapses in WT mice show Aβ-induced changes in postsynaptic glutamate receptor content, we explored whether there was any selectivity in the effect by studying synapses with left or right CA3 input and we obtained three measures of NMDA receptor contribution in addition to selectively inhibiting GluN2B subunit-containing NMDARs. GluN2A and GluN2B subunits confer different kinetic properties on the NMDAR, which influences Ca^2+^ influx [33], and they also make unique intracellular associations. The GluN2B subunit C-terminal domain binds Ca^2+/^calmodulin-dependent kinase II (CaMKII) with high affinity [34], anchoring it in its active conformation [35], which is required for LTP [36]. This means that the physical presence of GluN2B subunit-containing NMDARs at the synapse is likely to be particularly important for LTP, irrespective of their contribution to Ca^2+^ influx [37]. Consequently, we focused on possible changes in the composition of synaptic NMDARs.

Our data further suggest that tau is required for the Aβ-induced effect on GluN2B-rich synapses, since neither the reduction in N/A ratio nor the change in the decay time constant was observed in electrically-stimulated or optogenetically-activated synapses from the left CA3 in *Mapt*^*−/−*^ mice. It does not appear that this difference is accounted for by basal differences in the content or distribution of NMDAR subunits between wild-type and *Mapt*^*−/−*^ mice, since these genotypes have comparable magnitude of Ro 25–6981 effect, and *Mapt*^*−/−*^ mice show left–right input-dependent asymmetric distribution of GluN2B subunit-containing NMDARs similar to WT mice [10]. Previous observations in WT mice show that the left/right hippocampal origin of CA3 fibres, but not ipsilateral or contralateral projection, determines spine morphology [13], postsynaptic receptor composition, and plasticity [10]. Our three-way ANOVA on the NMDA_Q/_AMPA_peak_ outcome measure in *Mapt*^−/−^ mice was consistent with these previous observations, and the lack of ipsilateral/contralateral effect on WT mice with and without Aβ together with the left/right injection effect suggests the lack of ipsilateral/contralateral effects, suggesting that it is the left CA3 hippocampal origin and the high GluN2B content that determine the synaptic vulnerability to Aβ.

The most parsimonious explanation for how GluN2B currents are diminished is that acute Aβ triggers the loss of GluN2B subunit-containing NMDARs at the postsynaptic density (PSD) possibly through internalization or movement to extrasynaptic sites. The lack of tau might uncouple the Aβ-induced NMDAR changes at the synapse and hence preserve b-LTP. *Mapt*^*−/−*^ mice do not show an Aβ-induced impairment of high frequency-induced LTP either [6].

The reduction in GluN2B subunit-containing NMDAR-mediated current could arise because Aβ increases tau phosphorylation [21], which in turn encourages aggregation of tau and its increased presence in the somatodendritic compartment [38]. Aβ-induced tau hyperphosphorylation and mis-sorting impairs axonal transport [39], and this can be prevented by acute inhibition of the tau kinase GSK-3 [40], or a reduction in tau itself [41]. However, there was no evidence for a presynaptic impairment that could account for our data since the paired pulse ratio did not change following Aβ exposure. Instead, it is possible that disruption of dendritic transport mechanisms could impair delivery and replacement of glutamate receptors postsynaptically. The number of GluN2B subunit-containing NMDARs would be particularly susceptible to impaired delivery since they undergo more frequent endocytosis [42].

A possible candidate for a pathway by which Aβ alters synaptic GluN2B content in a tau-dependent manner is via the tyrosine kinase fyn, which is targeted by tau to the dendrite under normal conditions [43]. Increased activity of fyn downstream of Aβ binding to the prion protein has been shown to cause tau pathology [44] and dendritic spine loss [45]. In support of such a pathway, acute Aβ-induced neuronal death in organotypic hippocampal slices was prevented in *Fyn*^*−/−*^ mice [46]. A lack of tau may prevent the increased activity of fyn at the synapse, and thus have a protective effect on N/A ratio and b-LTP, which we have observed here. Fyn phosphorylates the GluN2B subunit enhancing PSD-95 binding [47] and preventing receptor internalization [42]. Fyn overactivation following minutes of Aβ exposure induced a transient increase in surface NMDARs, which correlated with increased GluN2B Y1472 phosphorylation, and resulted in excitotoxicity. This was followed by a decrease in GluN2B phosphorylation with a time course of hours [45]. By preventing this initial Fyn-induced increase of GluN2B-containing NMDARs, the lack of tau in *Mapt*^*−/−*^ mice could provide a protective mechanism that maintains a normal N/A ratio. The aberrant activation of the pathway that mediates the decrease of NMDAR function, STEP tyrosine phosphatase, has also been reported to play a role in Aβ-induced reduction of NMDAR-mediated currents [48, 49] and cognitive deficits [49]. The 3xTg-AD mouse model exhibits reduced synaptosomal GluN2B content and increased activity of striatal-enriched phosphatase 61 (STEP_61_) [49]. STEP_61_ dephosphorylates the GluN2B Y1472 site, encouraging endocytosis of GluN2B subunit-containing NMDARs, but also decreases the activity of Fyn by its dephosphorylation of a regulatory tyrosine. Therefore, much remains to be investigated about the precise changes in kinase and phosphatase activity that occur at different stages of Aβ-induced pathology.

An alternative explanation for the reduction in synaptic NMDAR current is that it is a downstream change compensating for Aβ-induced excitotoxicity mediated by GluN2B subunit-containing NMDARs. This would explain the time-dependent effects of Aβ on surface GluN2B subunit-containing NMDARs [45]. Acute exposure to Aβ can trigger increased Ca^2+^ influx through GluN2B subunit-containing NMDARs, particularly at extrasynaptic sites [50], leading to excitotoxicity. Reduction or lack of tau prevents Aβ-induced NMDAR-dependent excitotoxicity [43] and pre-exposure to GluN2B subunit-selective NMDAR antagonists can also prevent the LTP deficit induced by acute exposure to Aβ [50–52].

The suggestion that Aβ can trigger GluN2B-mediated excitotoxicity has led to GluN2B subunit-selective NMDAR antagonists being considered as potential drugs in AD. The NMDAR antagonist memantine provides symptomatic relief to AD patients [53], and improves cognitive function in certain tests in animals by reducing the interference of irrelevant information [54]. Whilst the open channel blocker memantine primarily targets overactive extrasynaptic NMDARs due to its fast off-rate and low affinity [53], our data suggest that caution should be exercised over potential treatments targeted specifically at inhibiting GluN2B subunit-containing NMDARs as a method to reduce extrasynaptic over-activity. In particular, this could impact an already reduced synaptic GluN2B subunit-containing NMDAR population likely to be vital for plasticity and learning. Indeed, chronic inhibition of GluN2B subunit-containing NMDARs does not rescue Aβ-induced synapse loss nor learning and memory deficits but instead impairs cognitive function [55]. Instead, our findings suggest that drugs designed to protect the normal function of synaptic GluN2B subunit-containing NMDARs might be a possible therapeutic avenue.

## Methods

### Mice

Animal care and experimental procedures were conducted in accordance with U.K. Home Office regulations under the Animals (Scientific Procedures) Act of 1986 under appropriate personal and project licences held by the authors. Mice were housed in polycarbonate cages of 5–10 animals and had access to food and water ad libitum. Holding facilities were maintained at approximately 22 °C, 60–70% humidity, and with a 12-h light–dark cycle (7 a.m. to 7 p.m.).

For the voltage-clamp experiments, 2–6 month old male *Mapt*^*−/−*^ mice on a C57BL6-J background [56] and age-matched male C57BL6-J controls were used. For synaptic plasticity experiments, 5–8 week old mice of both genotypes were used. C57BL6-J mice were purchased from Charles River Laboratories (Margate, U.K.) and *Mapt*^*−/−*^ mice were bred in-house. All mice were housed in the same animal facility under the same conditions for at least two weeks before experiments or surgery commenced.

### Surgery

Channelrhodopsin2 (ChR2) was used to isolate the inputs to CA1 originating in the left or right CA3. hChR2(E123T/T159C) was fused in-frame to enhanced yellow fluorescent protein (eYFP) (Berndt et al., 2011) and driven by a CaMKIIα promoter. Adeno-associated viral particles of serotype 5 were produced by the Vector Core Facility at The University of North Carolina at Chapel Hill.

*Mapt*^−/−^ or wild-type mice (2–4 months old) were anesthetized with 2–4% isoflurane at 0.6–1.4 L min^−1^. Using a stereotactic apparatus (Kopf Instruments, Tujunga, U.S.A.), the head was levelled and a small craniotomy was made 2.3 mm anterior and 2.2 mm lateral (either left or right) from the skull surface at bregma. Through a small durotomy, 0.6 μL virus suspension (AAV5-CaMKIIα-ChR2(E123T/T159C)- eYFP, 1–4 × 10^12^ viral molecules mL^−1^; University of North Carolina Vector Core, U.S.A.) were delivered at a rate of 0.1 μL min^−1^ 2.25 mm below the skull surface at bregma through a 33-gauge needle using a Hamilton Microliter syringe (Esslab, Hadleigh, U.K). Following a five-minute wait after bolus injection, the needle was retracted by 0.2 mm and after another five minutes slowly retracted fully. The scalp incision was sutured, and post-injection analgesic (0.03 mg kg^−1^ buprenorphine) was administered intraperitoneally to aid recovery. Following surgery, mice were left for 1–2 months for expression to develop. We injected 6 Mapt^−/−^ mice (3 left-injected/ 3 right-injected) and 24 WT mice (12 left-injected/ 12 right-injected) and recorded from either the left or right hippocampus. See Table 2 for numbers of cells recorded in each condition.

### Slice preparation and storage

Mice were deeply anesthetized by inhalation of isoflurane and decapitated. The brain was swiftly removed into ice-cold (0 to 1 °C) artificial cerebral-spinal fluid (aCSF) containing (in mM): 126 NaCl, 3 KCl, 1.25 NaH_2_PO_4_, 2 MgSO_4_, 2 CaCl_2_, 26 NaHCO_3_, and 10 glucose, pH 7.2–7.4.) bubbled with carbogen gas (95% O_2_ and 5% CO_2_). Slices were cut with a vibratome (Leica VT 1200S) in ice-cold aCSF. Coronal slices (350 μm) were used in optogenetic experiments and parasagittal hippocampal slices (350–400 μm) were used in all other experiments. After sectioning, slices were transferred to a submerged-style holding chamber at room temperature (22–27 °C) for at least one hour and then incubated in drug solutions when applicable.

### Electrophysiological protocols

Slices were transferred to a submerged-style recording chamber at room temperature and superfused with aCSF at 1–2 mL min^−1^. Whole-cell voltage-clamp recordings were performed with glass pipettes (3 to 5 MΩ for voltage clamp and 5 to 8 MΩ for current clamp) pulled from standard borosilicate glass. In voltage-clamp experiments, the intrapipette solution contained (in mM): CsCH_3_SO_3_ 120; CsCl 20; EGTA 0.2; HEPES 10.0; ATP-Mg 2.0; GTP 0.3; QX-314 10.0, adjusted to pH 7.2–7.3; osmolarity 285–300 mosmol L^−1^). In current-clamp experiments, the intrapipette solution contained (in mM): 110 potassium-gluconate, 40 HEPES, 2 ATP-Mg, 0.3 GTP, 4 NaCl (pH 7.2–7.3; osmolarity 270–290 mosmol L^−1^).

Cells with a pyramidal-shaped soma in the stratum pyramidale of CA1 were selected for recording using infrared, differential interference contrast optics. Voltage-clamp recordings were not started until at least 10 min after breakthrough to allow diffusion of Cs^+^ into the dendrites for improved space clamp. For voltage-clamp recordings, excitatory postsynaptic currents (EPSCs) were evoked either by a 50 μs pulse (80–300 μA) delivered by an extracellular stainless steel electrode (5 MΩ; A-M Systems) connected to a stimulus isolator unit (DS3, Digitimer Ltd., Welwyn Garden City, U.K.) or by a 100 μs pulse of blue laser light (473 nm, 1–5 mW at objective entry; Rapp OptoElectronic, Hamburg, Germany). The laser was coupled to the microscope with a 50 μm fiber (0.22 NA). Stimulation strength was adjusted to yield 100–200 pA EPSC peak amplitude at a holding potential of − 70 mV. EPSCs were evoked in the stratum radiatum every 14 s, alternating between 3 s steps at holding potentials of − 70 mV and + 65 mV, and returning to − 70 mV in between. To measure the paired-pulse ratio, two 50 μs pulses with an inter-pulse interval of 40 ms were given at a low stimulation strength and the maximum EPSC amplitude of each response was measured. The liquid junction potential of approximately − 15 mV was not corrected for. Series resistance was not corrected for but was monitored with test pulses continuously during recordings and a range of 10–20 MΩ was used. Cells in which the series resistance rose above 25 MΩ were not considered for analysis and recordings were rejected if the series resistance changed by more than 25%. Slices with polysynaptic responses were rejected. Recordings were made with an Axon Multiclamp 700B amplifier (Molecular Devices, Sunnyvale, U.S.A.). Signals were low-pass filtered at 2 kHz and acquired at 20 kHz using the Matlab acquisition software (Mathworks, Natick, U.S.A.) and custom software (MatDAQ, Hugh P.C. Robinson 1995–2013).

For current-clamp recordings, all cells were tested for regular spiking responses to positive current injection. During recordings, current injection was used to maintain cells at − 70 ± 3 mV. Excitatory postsynaptic potentials (EPSPs) were evoked at 0.2 Hz in the test and control pathway by a 50 μs pulse (100–350 μA) delivered by an extracellular stainless steel electrode (5 MΩ; A-M Systems). Pathway independence was confirmed by the lack of cross paired-pulse interactions when sequentially stimulating the two pathways with a 40 ms interval. A ten-minute baseline was recorded to minimize intracellular wash-out. Burst timing-dependent LTP was induced by pairing electrical stimulation followed 8 ms later by a postsynaptic burst of three action potentials, and this was repeated 100 times at baseline frequency. Following the burst-pairing protocol, which was only applied to the test pathway, EPSPs were recorded in both pathways for a further 35 min. EPSP slope was reported relative to the average of the last 5 min of baseline recording. Input resistance (R_in_) was monitored throughout the duration of the experiment, and cells were rejected if the R_in_ changed by more than 20%. Recordings were made with an Axon Multiclamp 700B amplifier (Molecular Devices). Data were filtered at 2 kHz and acquired at 5 kHz using an Instrutech data acquisition board and custom-made procedures in IGOR Pro (Wavemetrics, Oregon, USA).

### Data analysis

Analysis of LTP data was performed using custom-written procedures in IGOR Pro (Wavemetrics). Synaptic efficacy was assessed from the EPSP slope, measured on the rising phase of the EPSP as a linear fit between time points that corresponded to 30% and 60% of the peak amplitude during the baseline. The post pairing EPSP slopes were normalized to the mean EPSP slope during the 10 min of baseline recording. For statistical comparisons, the mean EPSP slope between 30 and 35 min after LTP induction was used. Representative traces of EPSPs are an average of 12 consecutive recordings.

Paired pulse values (measured at − 70 mV) were obtained by dividing the peak amplitude of the second EPSC by the peak amplitude of the first EPSC; reported values are the means of 5 individual paired pulse values. Representative traces of EPSCs are an average of 10 consecutive recordings.

Measurements of AMPAR and NMDAR-mediated currents were made using custom-written procedures in Matlab (Mathworks). The NMDAR charge transfer was calculated by integrating the NMDAR current from 2.5 to 1250 ms after synaptic stimulation. The AMPAR-mediated current measurement was made using the peak current recorded at a holding potential of -70 mV. For NMDAR-mediated current measurements, cells were depolarized to + 65 mV for 2 s prior to synaptic stimulation. Leak corrected currents were analyzed. For N/A ratios, the average NMDAR current was measured 55–57 ms after synaptic stimulation. The average value of NMDAR- and AMPAR-mediated current was calculated per cell, and a normalized N/A ratio given by dividing the NMDA value by the AMPAR value. The average current trace recorded at + 65 mV was fitted using a least square method (Matlab) to the following double exponential function equation:

$$I\left( t \right) = I_{f} e^{{ - t/\tau_{f} }} + I_{s} e^{{ - t/\tau_{s} }}$$

where *I*_*f*_ and *I*_*s*_ are the amplitudes of the fast and slow component, t = time, and $\tau_{f}$ and $\tau_{s}$ are the fast and slow time constants, respectively. The weighted decay time constant $(\tau_{w} )$ was computed by using the fitted values as follows [57]

$$\tau_{w} = \left[ {I_{f} /(I_{f} + I_{s} )} \right] \tau_{f} + \left[ {I_{s} /(I_{f} + I_{s} )} \right] \tau_{s}$$

Statistical analyses were performed using SPSS or Matlab and statistical significance was assessed by two or three-way ANOVA as indicated. Post hoc comparisons were performed using Student’s one-tailed unpaired *t*-tests to test for reduction in our outcome measures as per our hypotheses. Bonferroni post-hoc correction was used in Figs. 1 and 2. In Table 1, the Benjamini-Hochberg procedure was used for controlling the false discovery rate (FDR) in our family of hypothesis tests. All values are given as mean ± SEM, and numbers (*n*) refer to the number of cells. Percentage reduction and error were calculated with standard error propagation. All data presented together in the same figure were performed interleaved between genotypes and experimental conditions. Error bars represent SEM.

### Pharmacology

In all voltage-clamp recordings, SR 95,531 hydrobromide (gabazine) (R&D Systems, Abingdon, UK) was included in the aCSF at a concentration of 3 μM (prepared from 6 mM frozen stock dissolved in water). Afferents from CA3 were cut before the slice was transferred into the recording chamber. Ro 25–6981 maleate (R&D Systems) was prepared as a stock solution of 5 mM with water and stored in frozen aliquots. Single aliquots were defrosted on the day of use and diluted to the final concentration of 0.5 μM in aCSF.

**Fig. 4:**
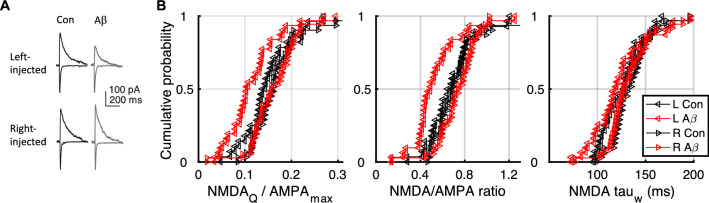
Human Aβ_1-42_ reduces NMDAR current in left, but not right, CA3-CA1 synapses in wild-type mice. **a** Sample traces from currents recorded in hippocampal CA1 neurons after optogenetic activation of axons originating in left or right CA3. Currents in control conditions and after Aβ incubation are presented. **b** Cumulative distribution plot showing synaptic NMDAR charge transfer (NMDA_Q_/AMPA_max_), N/A ratio, and weighted NMDAR current decay time constant (τ_w_) in synapses activated by inputs from left CA3 or right CA3, showing a selective inhibition (shift to the left) by hAβ_1-42_ in synapses with input from the left CA3 only. Each point represents the average value per cell. Statistical comparisons are presented in Table 1

Human Aβ_1-42_ (hAβ_1-42_) was freshly prepared on the day of experiment. Data in Figs. 2, 3, 4 were collected using synthetic hAβ_1-42_ from R&D Systems, prepared as described in [6]. Briefly, hAβ_1-42_ was initially dissolved in aCSF to a concentration of 5 mM; aliquots were then sonicated for 11 min before final dilution to 220 nM in aCSF. Hippocampal slices were incubated in a submerged-style holding chamber in aCSF with or without hAβ_1-42_ for 1–3 h before recording. Superfusion with half concentration of the drug continued after slices were transferred to the recording chamber. Data in Fig. 1 were collected using hAβ_1-42_ (AggreSure) from AnaSpec (CA, USA); this was initially reconstituted in a buffer solution containing 20 mM HEPES and 150 mM NaCl at 0.25 mg/mL, aliquots were then sonicated for 11 min and incubated at 37 ± 2 ºC for 60 min with gentle shaking before being diluted to a final concentration of 275 nM in aCSF. Hippocampal slices were incubated in a submerged-style holding chamber in aCSF with hAβ_1-42_ or a control buffer solution (100 µM HEPES and 750 µM NaCl) for 1–3 h before recording.

## Data Availability

The datasets analyzed in the current study are available from the corresponding authors on reasonable request.
